# Exogenous Methyl Jasmonate Treatment Increases Glucosinolate Biosynthesis and Quinone Reductase Activity in Kale Leaf Tissue

**DOI:** 10.1371/journal.pone.0103407

**Published:** 2014-08-01

**Authors:** Kang-Mo Ku, Elizabeth H. Jeffery, John A. Juvik

**Affiliations:** 1 Department of Crop Sciences, University of Illinois at Urbana-Champaign, Urbana, Illinois, United States of America; 2 Department of Food Science and Human Nutrition, University of Illinois at Urbana-Champaign, Urbana, Illinois, United States of America; Kobe University, Japan

## Abstract

Methyl jasmonate (MeJA) spray treatments were applied to the kale varieties ‘Dwarf Blue Curled Vates’ and ‘Red Winter’ in replicated field plantings in 2010 and 2011 to investigate alteration of glucosinolate (GS) composition in harvested leaf tissue. Aqueous solutions of 250 µM MeJA were sprayed to saturation on aerial plant tissues four days prior to harvest at commercial maturity. The MeJA treatment significantly increased gluconasturtiin (56%), glucobrassicin (98%), and neoglucobrassicin (150%) concentrations in the apical leaf tissue of these genotypes over two seasons. Induction of quinone reductase (QR) activity, a biomarker for anti-carcinogenesis, was significantly increased by the extracts from the leaf tissue of these two cultivars. Extracts of apical leaf tissues had greater MeJA mediated increases in phenolics, glucosinolate concentrations, GS hydrolysis products, and QR activity than extracts from basal leaf tissue samples. The concentration of the hydrolysis product of glucoraphanin, sulforphane was significantly increased in apical leaf tissue of the cultivar ‘Red Winter’ in both 2010 and 2011. There was interaction between exogenous MeJA treatment and environmental conditions to induce endogenous JA. Correlation analysis revealed that indole-3-carbanol (I3C) generated from the hydrolysis of glucobrassicin significantly correlated with QR activity (*r* = 0.800, *P*<0.001). Concentrations required to double the specific QR activity (CD values) of I3C was calculated at 230 µM, which is considerably weaker at induction than other isothiocyanates like sulforphane. To confirm relationships between GS hydrolysis products and QR activity, a range of concentrations of MeJA sprays were applied to kale leaf tissues of both cultivars in 2011. Correlation analysis of these results indicated that sulforaphane, NI3C, neoascorbigen, I3C, and diindolylmethane were all significantly correlated with QR activity. Thus, increased QR activity may be due to combined increases in phenolics (quercetin and kaempferol) and GS hydrolysis product concentrations rather than by individual products alone.

## Introduction

Epidemiological studies have reported that the intake of *Brassica* vegetables is inversely correlated with cancer risk, and this association is stronger than those between cancer and fruit and vegetable consumption in general [1]. Kale (*Brassica oleracea* L. *acephala*) is a frequently consumed leafy vegetable. Young tender leafs are harvested for human consumption and older plant tissues for animal feed [2]. Kale is a good source of vitamins (Vitamin A, C, and E) and of health promoting phytochemicals including glucosinolates (GS), carotenoids, phenolics, and tocopherols. In certain regions like on the Iberian Peninsula, kale (*Brassica oleracea acephala* group) leaves and flower buds are grown and harvested for consumption throughout the year [2].

There are several types of kales. Among them, it was previously reported that GS composition of Siberian kale (*B. napus*) was distinct from ‘Vates’ (*B. oleracea*) type kale [3]. Red Russian and Siberian kales *(Brassica napus ssp. pabularia)* are typically more tender and have a milder flavor than the European “oleracea” kales whose young leaves are superior for use in salads. Napus kales have good cold tolerance so that they can be grown anywhere in the US over a broader range of growing seasons and are also used as animal forage. Forage and root vegetable cultivars of *B. napus* show high levels of progoitrin [4] which can promote goitrogenic effects in mammals [5]. Although cultivars of *Brassica napus* are thought to have originated from a chance hybridization between *Brassica rapa* and *Brassica oleracea*, the Red Russian type of kales were bred by artificial hybridization (http://seedambassadors.org/Mainpages/still/napuskale/napuskale.htm). The ‘Red Winter’ cultivar was derived from Red Russian kale types.

*B. oleracea* kale is a rich source of flavonoids, possessing up to 47 mg of kaempferol and 22 mg of quercetin per 100 g of fresh leaf tissue. Kale contains the highest flavonoid content among all of the *Brassica oleracea* vegetables [6]. Phenolics have putative antioxidant, anticancer, and anti-cardiovascular disease activity [7]–[9]. Previous research revealed that MeJA treatments enhance total polyphenolic compounds and flavonoids in kale leaf tissues [10]. The response to MeJA treatment was more dramatically observed in young tissue (apical leaves) compared to old tissue (basal leaves) [10].

Besides phenolic compounds, kale is also good source of GS. Glucosinolates are a class of secondary metabolites found in cruciferous crops. The breakdown products have been shown to affect human health, insect herbivory, and plant resistance to pathogens [11]–[13]. Some GS breakdown products have a chemoprotective effect against certain cancers in humans [14].

Up-regulation of phase II enzyme detoxification activity has been suggested as a good strategy for cancer prevention [15], [16]. Phase II detoxifying enzymes including glutathione S-transferase (GST) and quinone reductase (QR) can enhance detoxification and elimination of carcinogens from the body [15], [16]. Hydrolysis products of GS, isothiocyanates such sulforaphane and phenethyl isothiocyanate (PEITC) have been shown to enhance quinone reductase (QR) and provide other chemopreventive activities [17], [18]. Previous studies have reported that the hydrolysis products of the indolyl GS including glucobrassicin and neoglucobrassicin also have cancer chemopreventive activity. Hydrolysis products of glucobrassicin including indole-3-carbinol (I3C), diindolylmethane, and ascorbigen induce QR [17], [19]. *N*-methoxyindole-3-carbinol (NI3C) and neoascorbigen (NeoASG), the hydrolysis product of neoglucobrassicin has been reported to induce cell cycle arrest in human colon cancer cell lines [20] and to induce QR activity [21].

The GS are also associated with insect defense in *Brassica* species. Jasmonic acid (JA), an endogenous plant signal transduction compound whose biosynthesis is up-regulated when *Brassica* plant species are attacked by herbivores, causes enhanced indolyl GS biosynthesis [22]. The increased GS concentrations induced by exogenous MeJA spray treatment was found to be a species-specific response [23]. MeJA treatment significantly increased gluconasturtiin and neoglucobrasicin in broccoli [24] and glucoraphanin, glucobrassicin, and neoglucobrassicin in cauliflower [23]. In addition, MeJA treatment significantly increased QR inducing activity and nitric oxide production inhibitory activity in broccoli and cauliflower [21], [23], [25], [26]. To date, GS compositional changes of kale leaf tissue induced by exogenous MeJA treatments have not been previously reported in the literature.

Compared to other *Brassica* vegetables including broccoli, watercress, and Brussels sprouts, anti-cancer bioactivity information about kale is limited [18], [19], [27]. The objective of this research is to determine the QR inducing health promoting effect derived from elevated phytochemical concentrations induced by MeJA in two different kale types.

## Materials and Methods

### Plant Cultivation

The cultivars ‘Red Winter’ (RW, *Brassica napus ssp. pabularia)* and ‘Dwarf Blue Curled Vates’ (DBCV, *Brassica oleracea* L. var. *acephala*) used for these experiments were purchased from Burpee Seed Co. (Warminster, PA). Seeds of each kale genotype were germinated in 32 cell plant plug trays filled with sunshine LC1 (Sun Gro Horticulture, Vancouver, British Columbia, Canada) professional soil mix. Seedlings were grown in a greenhouse at the University of Illinois at Urbana-Champaign under a 25°C/15°C and 14 h/10 h: day/night temperature regime with supplemental lighting. Thirty days after germination, seedling trays were placed in a ground bed to harden off for a week prior to transplanting into field plots at the University of Illinois South Farm (40° 04′38.89″ N, 88° 14′26.18″ W). Experimental design was a split-plot arrangement in a randomized complete block (RCB) design with three replicates. The experimental plot was surrounded by a guard row to avoid border effects. Transplanting of kale seedlings was conducted on June 11, 2010 and June 13, 2011. Harvesting kale occurred on July 25 in 2010 and July 27 in 2011. Irrigation was only applied during the first week of cultivation for the establishment of transplanted seedlings. Weather conditions during the 2010 and 2011 growing seasons collected from Illinois State Water Service (http://www.isws.illinois.edu/warm/data/cdfs/cmiday.txt) are presented in Table 1.

**Table 1:** Weather information during the growing seasons of 2010 and 2011 for Champaign, Illinois.

	Total solar radiation (MJ·m^−2^)		
Year	Jun	Jul	Sum
2010	720	730	1450
2011	667	790	1457
% of (2011/2010)	93	108	100.5
	**Precipitation (mm)**		
Year	Jun	Jul	Sum
2010	199	91	290
2011	107	40	147
% of (2011/2010)	54	44	50.7
	**Growing degree days (°C)**		
Year	Jun	Jul	Sum
2010	373	408	781
2011	362	430	792
% of (2011/2010)	97	105	101.4

### Kale Treatment with MeJA and Sample Preparation

**Figure 1:**
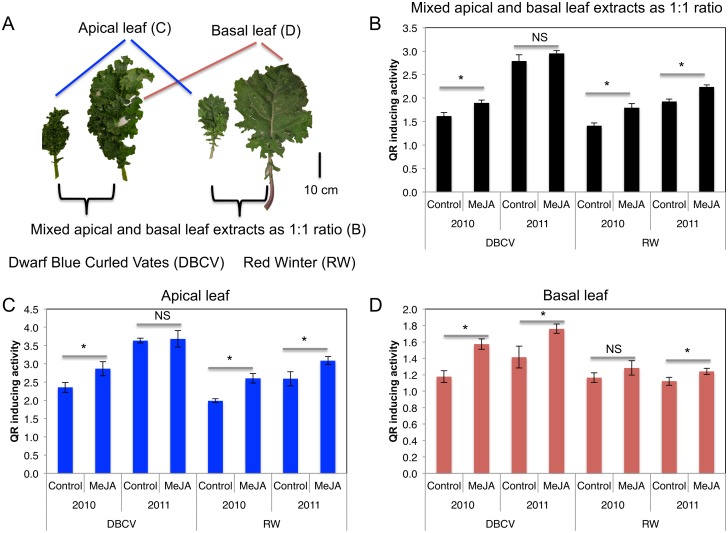
QR inducing activity of apical, basal and combined leaf tissue samples from two kale cultivars. A: Images of harvested apical and basal leaf samples. B: QR activity of mixed extract of 1∶1 apical and basal leaf tissues. C: QR activity of apical leaf tissue. D: QR activity of basal leaf tissue. Student T-tests were conducted to determine significance at *P*≤0.05. NS and *indicate non-significance and significance at *P*≤0.05, respectively. Data are means ± SD (n = 3).

An aqueous solution of 250 µM MeJA (Sigma-Aldrich, St. Louis, MO) and 0.1% Triton X-100 (Sigma-Aldrich) was sprayed on all aerial plant tissues to the point of runoff (approximately 100 mL) four days prior to harvest based on the result of experiments to determine when GS levels are optimized prior to harvest (Figure S1). Two different kale leaf samples (apical: three leaves from the below the meristematic growing point, at a minimum 8 cm in length; basal: three fully expanded leaves nearest the soil surface without discoloration or signs of senescence or damage) were harvested and bulked from five treated and control plants of each genotype for each replicate respectively (five leaves bulked for a replicate sample). Images of apical and basal samples of each kale cultivar are shown in Figure 1A. In order to confirm the relationship between increased hydrolysis products of GS and QR activity, 0, 50, 250, and 500 µM MeJA were sprayed on kale leaf tissue as described above in 2011. All kale leaf tissue samples were frozen in liquid nitrogen, and stored at −20°C prior to freeze-drying. Freeze-dried tissues were ground into a fine powder using a coffee grinder and stored at −20°C prior to chemical and bioactivity analyses.

### Quinone Reductase (QR) Inducing Activity

Freeze-dried kale leaf powder (75 mg) was suspended in 1.5 mL of water in the absence of light for 4 h (time for the maximum concentration of indolyl GS hydrolysis products) at room temperature in a sealed 2 mL microcentrifuge tube (Fisher Scientific, Waltham, MA) to facilitate GSs hydrolysis by endogenous myrosinase. Slurries were then centrifuged at 12,000×g for 5 min and supernatants was used for QR assay. QR inducing activities were measured for individual apical and basal leaf tissue extracts and a pooled equal volume sample from both apical and basal leaf tissue extracts (Figure 1A). Hepa1c1c7 murine hepatoma cells (ATCC, Manassas, VA) were grown in alpha-minimum essential medium (α-MEM), enriched with 10% fetal bovine serum and maintained at 37°C in 95% ambient air and 5% CO_2_. The cells were divided every three days with a split ratio of 7. Cells with 80–90% confluence were plated into 96-well plates (Costar 3595, Corning Inc, Corning, NY), 1×10^4^ cells per well, and incubated for 24 h in antibiotic-enriched media (100 units/mL penicillin, 100 µg/mL streptomycin). The QR induction activities of different samples were determined by means of the protocol described by Prochaska & Santamaria [28]. After 24 h cells were exposed to the different sample extracts [0.25% final concentration (125 µg of freeze-dried kale/mL) in 200 µL of media] in new media for a further 24 h. Growth media alone was used as a negative control. Treated cells were rinsed with phosphate buffer at pH 7.4, lysed with 50 µL 0.8% digitonin in 2 mM EDTA, incubated and agitated for 10 min. A 200-µL aliquot of reaction mix [10 µM BSA, 82 µM Tween-20 solution, 927 µM glucose-6-phosphate, 1.85 µM NADP, 57 nM FAD, 2 units of glucose-6-phosphate dehydrogenase, 725 nM 3-(4,5-dimethylthiazo-2-yl)-2,5-diphenyltetrazolium bromide (MTT), and 50 µM menadione (dissolved in acetonitrile) in 25 mM Tris buffer] was added to the lysed cells. Readings were made at five time points, 50 s apart, using a µQuant microplate reader (Bio-Tek Instruments, Winooski, VT) at 610 nm. Immediately after completion of the readings, 50 µL of 0.3 mM dicumarol in 25 mM Tris buffer was added into each well, and the plate was read again (five time points, 50 s apart) to determine non-specific MTT reduction. Total protein content was measured by the BioRad assay (Bio-Rad, Hercules, CA) using manufacture’s instructions. Activity was expressed as QR specific activity (nmol MTT reduced/mg/min) ratio of treated to negative control cells. In order to measure QR inducing activity associated with phenolic compounds in extracts, freeze-dried kale powder (0.2 g) and 4 mL of 70% methanol were added to 10 mL tubes (Nalgene, Rochester, NY) and heated on a heating block at 95°C for 10 min. After cooling on ice, the extract was centrifuged at 3,000×g for 10 min at 4°C. The supernatant (1 mL) was dried up using SpeedVac (Savant, Osterville, MA) and reconstituted with DMSO (1 mL). QR inducing activity of phenolic rich-extract was measured using the same procedures and concentrations described above.

### Determination of Sample GS Concentrations

Extraction and quantification of GS using high-performance liquid chromatography was performed using a previously published protocol [29]. Freeze-dried kale powder (0.2 g) and 2 mL of 70% methanol were added to 10 mL tubes (Nalgene) and heated on a heating block at 95°C for 10 min. After cooling on ice, 0.5 mL benzylglucosinolate (1 mM) was added as internal standard (POS Pilot Plant Corp, Saskatoon, SK, Canada), mixed, and centrifuged at 3,000×g for 10 min at 4°C. The supernatant was saved and the pellet was re-extracted with 2 mL 70% methanol at 95°C for 10 min and the two extracts combined. A subsample (1 mL) from each pooled extract was transferred into a 2-mL microcentrifuge tube (Fisher Scientific, Waltham, MA). Protein was precipitated with 0.15 mL of a 1∶1 mixture of 1 M lead acetate and 1 M barium acetate. After centrifuging at 12,000×g for 1 min, each sample was then loaded onto a column containing DEAE Sephadex A-25 resin (GE Healthcare, Piscataway, NJ) for desulfation with arylsulfatase (*Helix pomatia* Type-1, Sigma-Aldrich, St. Louis, MO) for 18 h and the desulfo-GS eluted. Samples (100 µL) were injected on to an Agilent 1100 HPLC system (Agilent, Santa Clara, CA), equipped with a G1311A bin pump, a G1322A vacuum degasser, a G1316A thermostatic column compartment, a G1315B diode array detector and an HP 1100 series G1313A autosampler. UV detector set at 229 nm wavelength. All-guard cartridge pre-column (Alltech, Lexington, Kentucky), and a LiChospher 100 RP-18 column (Merck, Darmstadt, Germany) were used for quantification. Desulfo-GS were eluted from the column over 45 min with a linear gradient of 0% to 20% acetonitrile at a flow rate of 1 mL/min. Benzylglucosinolate was used as an internal standard and UV response factors for different types of GS were used as determined by previous study [30]. The identification of desulfo-GS profiles were validated by LC-tandem MS using a Waters 32 Q-Tof Ultima spectrometer coupled to a Waters 1525 HPLC system and full scan LC-MS using a Finnigan LCQ Deca XP, respectively. The molecular ion and fragmentation patterns of individual desulfo-GS were matched with the literature for GS identification [31], [32].

### Analysis of endogenous JA in kale leaf tissues

Endogenous JA concentrations in kale leaf tissues were measured using a previously published method [33]. Samples (100 mg) of freeze-dried kale leaf powder were extracted with 1. 5 mL methanol–water–acetic acid (90∶9∶1, v/v/v) and centrifuged for 1 min at 10,000 rpm. The supernatant was collected and the extraction repeated. Pooled supernatants were dried under N_2_, resuspended in 200 µL of 0.05% acetic acid in water–acetonitrile (85∶15, v/v), and filtered with a Millex-HV 0.45 µm filter from Millipore (Bedford, MA). Quantitation was estimated using external standards of a range of JA solutions (ranging from 1.25 to 10 pM). Analyses were carried out using a LC-tandem MS using a Waters 32 Q-Tof Ultima spectrometer coupled to a Waters 1525 HPLC system. All the analyses were performed using negative ion mode with a collision energy (CE) of −25 V. MRM acquisition was done by monitoring the 209/59 transitions for JA. An Eclipse XDB-C18 column (150×4 mm, particle size 5 µm, Agilent, Santa Clara, CA) was used at ambient temperature and the injected volume was 10 µl. The elution gradient was carried out with a binary solvent system consisting of 0.05% acetic acid in water (solvent A) and acetonitrile (solvent B) at a constant flow-rate of 0.6 mL/min. A linear gradient profile with the following proportions (v/v) of solvent B was applied (t (min), %B): (0, 15), (3, 15), (5, 100), (6, 100), (7, 15), (8, 15) with 5 min for re-equilibration.

### Determination of Total Myrosinase Activity Using Glucose Release

Total myrosinase activity was measured with whole kale tissue using the ABTS-glucose assay [26] without protein extraction to avoid introducing any artifacts. Freeze-dried kale (50 mg) was weighed in duplicate into 2 mL tubes and one mL sinigrin (10 mM, Sigma) was added to each tube. After 10 s of vigorous vortexing, one of the paired samples was put directly into a heating block (95°C) for 10 min to inactivate the myrosinase enzyme (zero time blank). The second sample was incubated at 40°C for 30 min and then inactivated as outlined above. After inactivation, samples were cooled on ice for 5 min then centrifuged at 16,000 g for 2 min. The supernatants were diluted 96-fold and aliquots (30 µL) or glucose standards were added in a 96 well plate and followed by adding 200 µL of an ABTS-glucose solution [2.7 mM ABTS, 1,000 units peroxidase (Type VI-A, Sigma), and 1,000 units glucose oxidase in 100 mL], incubated for 20 min and absorbance measured at 630 nm in a µQuant plate reader (Bio-Tek instruments, Winooski, VT).

### Analysis of Glucosinolate Hydrolysis Products

The extraction and analysis of isothiocyanates and other hydrolysis products was carried out according to previously published methods [21], [26], [34]. For the GS hydrolysis products, kale extracts were collected using the same protocol for the QR assay described above with sampling at 4 h and 24 h of incubation, which are hydrolysis duration periods that generate maximum concentrations for indolyl GS products and sulforaphane, respectively [21]. Freeze-dried kale leaf powder (75 mg) was suspended in 1.5 mL of water in the absence of light for 4 h and 24 h at room temperature in a sealed 2 mL microcentrifuge tube (Fisher Scientific, Waltham, MA) to facilitate GS hydrolysis by endogenous myrosinase. Slurries were then centrifuged at 12,000×g for 5 min and 0.5 mL of supernatants was transferred into a 2 mL microcentrifuge tube. Butyl isothiocyanate (0.5 mg/mL) and 4-methoxyindole (1 mg/mL) solutions were prepared and mixed in a 1∶1 (v/v) ratio. An aliquot of this solution (40 µL) was added as the internal standards for sulforphane and the hydrolysis products of indolyl GS (I3C, DIM, NI3C, and NeoASG), to quantify respectively. After adding 0.5 mL of methylene chloride, tubes were shaken vigorously before being centrifuged for 2 min at 9,600 g. The methylene chloride layer (200 µL) was transferred to 350 µL flat bottom insert (Fisher Scientific, Pittsburgh, PA) in a 2 mL HPLC autosampler vial (Agilent, Santa Clara, CA) for mixing with 100 µL of a reagent containing 20 mM triethylamine and 200 mM mercaptoethanol in methylene chloride. The mixture was incubated at 30°C for 60 min under constant stirring, and then dried under a stream of nitrogen. The residue containing isothiocyanate derivatives (isothiocyanate-mercaptoethanol derivatives) and other hydrolysis compounds was dissolved in 200 µL of acetonitrile/water (1∶1) (v/v), and 10 µL of this solution injected onto a Agilent 1100 HPLC system (Agilent, Santa Clara, CA), equipped with a G1311A bin pump, a G1322A vacuum degasser, a G1316A thermostatic column compartment, a G1315B diode array detector and an HP 1100 series G1313A autosampler. Extracts were separated on an Eclipse XDB-C18 column (150×4 mm, particle size 5 µm, Agilent, Santa Clara, CA) with an Adsorbosphere C18 all-guard cartridge pre-column (Grace, Deerfield, IL). Mobile phase A was water and B methanol. Mobile phase B was 0% at injection, increasing to 10% by 10 min, 100% at 35 min, and held 5 min, then decreased to 0% by 50 min. Flow rates were kept at 0.8 mL/min. The detector wavelength was set at 227 and 271 nm. Response factors of monomeric indolyl derivatives were used from a previous report [35]. Due to a lack of standards for NI3C and NeoASG the standard curve of I3C was applied for quantification of both NI3C and NeoASG. The quantity was expressed as I3C equivalent concentrations.

### QR Inducing Activity Measurement of I3C

QR activity of hydrolysis product, I3C was measured to determine the concentrations required to double the specific activity of QR (CD value). Commercially purchased I3C (Sigma-Aldrich) was dissolved in DMSO, then seven concentrations (250, 125, 62.5, 31.3, 15.6, 7.8, and 3.9 µM) of I3C prepared by serial two fold dilutions and added to 96 well plates of cultured hepa1c1c7 cells. After 24 h incubation, QR activity was measured using the protocol described above.

### Statistical Analysis

Analysis of variance (ANOVA) was conducted using JMP 10 statistical software program (SAS institute Inc., Cary, NC). Year, treatments, and genotype effects were considered as fixed factors. Block was considered as random. Analysis of variance was performed using the linear model: Y_ijklm_ = m+G_i_+Y_j_+T_k_+GY_ij_+GT_ik_+YT_jk_+GYT_ijk_+B_l(j)_+ε_ijklm_, where Y_ijklm_ is the l^th^ block of the phenotypic value of the k^th^ treatment, i^th^ genotype in year j, m is the overall mean, G, Y, T, and B indicate the effects of genotype, year (weather), treatment and blocks nested in years, ε_ijklm_ is the experimental error associated with Y_ijklm_, respectively. Fisher’s Least Significant Difference (LSD) test, correlation analysis and Student’s t-tests were also conducted using the JMP 10 software. All sample analyses were conducted in triplicate. The results are presented as means ± SD.

## Results and Discussion

### Effect of MeJA Treatment on QR Inducing Activity of Kale Leaf Tissues

MeJA treatment significantly increased QR activity in the combined apical and basal leaf extracts of the two different kale species extracts over two years except for the DBCV cultivar in 2011 (Figure 1B). There was significant year-to-year variation in QR activity with 2011 samples significantly greater than those in 2010. In 2010 apical leaf tissue extracts of MeJA treated kale increased 17% and 27% over QR activity for DBCV and RW controls, respectively, while in 2011, they increased only by 6% and 16%. QR activities of apical leaf tissue extract were up to 2-fold greater than extracts from basal leaves (Figure 1C and 1D), which is of relevance to vegetable growers where kale is harvested throughout the year.

### Effect of MeJA Treatment on QR Inducing Activity Associated with Phenolic Rich-Extract of Kale Leaf Tissues

A previous study reported that MeJA treatment specifically increased phenolic and flavonoid concentrations in kale leaves primarily in the form of the flavonoids, quercetin and kaempferol [10]. In order to test if tissue phenolic concentrations induced by MeJA treatment contribute to QR inducing activity, we also measured QR inducing activity in phenolic rich-extracts after myrosinase inactivation by heating. Unlike aqueous extracts, there was no consistent QR activity increases associated with MeJA treatments in phenolic rich-extracts (Figure S2). The only significant increase was observed in apical leaf of RW in both years and basal leaf of DBCV in 2011. Aqueous kale extracts in this study have both GS hydrolysis products and water soluble phenolics. After subtracting QR inducing activity by phenolic rich-extracts from QR inducing activity by aqueous extract, we approximately calculated the contribution of phenolics to QR inducing activity. Averaged QR inducing activity derived by phenolic-rich, myrosinase-inactivated extracts of RW kale apical leaves accounted from 56% and 72% of the QR induction of aqueous extracts in 2010 and 2011, respectively. The phenolic rich-extracts of DBCV apical leaves accounted for 58% and 33% of QR inducing activity of aqueous extract in 2010 and 2011, respectively. Quercetin and kaempferol have been reported as QR inducers [36]. Also, glucoside forms of quercetin have been reported as QR inducers from onion [37]. Thus, it is possible that flavonoids in broccoli can contribute the QR induction. Since it is not feasible to completely inactivate myrosinase enzyme using water, we used 70% methanol to inactivate the enzyme. Using this different extraction solvent may lead to overestimating the contribution of phenolic compounds to QR activity because it can extract non-polar compounds as well. Nevertheless, this calculation suggests that phenolic compound concentrations induced by MeJA treatment partially contribute to QR inducing activity of kale leaf tissues. The magnitude of contribution is different based on the cultivar and year.

### Effect of MeJA Treatment on GS Concentrations

Over both seasons, MeJA treatments significantly increased glucobrassicin and neoglucobrassicin concentrations in both apical and basal leaves. The treatment increased apical leaf concentrations of gluconasturtiin (56%), glucobrassicin (98%), and neoglucobrassin (150%) and basal leaf concentrations of gluconasturtiin (44%), glucobrassicin (166%) and neoglucobrassin (83%) averaged across cultivars and over years (Table 2). Total GS concentration in apical leaf tissues was up to seven fold greater than basal leaf tissues. This concentration difference can explain why apical leaf extracts induced higher QR activity.

**Table 2:** GS composition of different kale leaf tissue samples with or without MeJA treatment from two kale cultivars over two years.

Apical tissue	––––––––DBCV––––––––				––––––––RW––––––––			
	2010		2011		2010		2011	
(µmol/g DW)	Control	MeJA	Control	MeJA	Control	MeJA	Control	MeJA
Glucoiberin	2.10	2.70^ns^	9.62	9.20^ns^	0.03	0.08^ns^	0.00	0.04^ns^
Progoitrin	0.12	0.04^ns^	0.90	0.39^ns^	22.02	22.97^ns^	31.59	30.53^ns^
Glucoraphanin	0.32	0.21^ns^	0.37	0.32^ns^	2.28	2.14^ns^	5.48	3.52^ns^
Gluconapin	0.61	0.85^ns^	1.79	1.25^ns^	0.98	0.81^ns^	1.22	2.05^ns^
Glucobrassicin	9.32	25.52*	22.44	29.56*	9.62	19.24*	5.93	19.50*
Gluconasturtiin	0.47	1.14*	1.22	1.44^ns^	2.40	3.48*	2.05	3.57^ns^
Neoglucobrassicin	2.30	9.66*	6.01	10.15*	7.88	20.72*	6.17	15.35*
**Basal tissue**	**––––––––DBCV––––––––**				**––––––––RW––––––––**			
	**2010**		**2011**		**2010**		**2011**	
**(µmol/g DW)**	**Control**	**MeJA**	**Control**	**MeJA**	**Control**	**MeJA**	**Control**	**MeJA**
Glucoiberin	0.32	0.43^ns^	0.71	0.81^ns^	0.02	0.04^ns^	0.00	0.00^ns^
Progoitrin	0.30	0.10^ns^	0.05	0.05^ns^	1.19	2.04^ns^	2.14	2.33^ns^
Glucoraphanin	0.17	0.22^ns^	0.11	0.12^ns^	0.13	0.14^ns^	0.05	1.02^ns^
Gluconapin	1.05	0.43^ns^	1.22	0.93^ns^	0.19	0.31*	1.26	1.16^ns^
Glucobrassicin	1.65	6.53*	2.53	4.54*	0.63	2.04*	0.61	1.36*
Gluconasturtiin	0.36	0.80^ns^	0.47	0.53^ns^	0.34	0.48*	0.35	0.36^ns^
Neoglucobrassicin	0.69	1.67*	0.97	1.04*	1.04	2.56*	1.71	2.79*

Student T-tests were conducted to determine significance between control and MeJA treatment at *P*≤0.05. ^ns^ and *indicate non-significance and significance at *P*≤0.05, respectively. Data are mean value from triplicates.

From previous work, *B. napus* type kales (such as RW) have distinct GS compositional profiles compared with *B. oleracea* type kale [3]. As Figure S1 and Table 2 illustrate, the major GS in both DBCV and RW are glucobrassicin and neoglucobrassicin. However, DBCV contains a higher concentration of glucoiberin while RW is higher in progoitrin. Unlike DBCV, RW contains relatively high glucoraphanin concentrations.

MeJA mediated enhancement of GS concentrations in DBCV was greater in 2010 than in 2011 where glucobrassicin and total GS concentrations in apical leaf tissues were both 2.7 fold higher in 2010 compared to increases of 1.3 and 1.2 fold, respectively in 2011 (Table 2). MeJA treatments may be interacting with varying weather conditions in each season of application. This variation may be associated with reduced rainfall in 2011, which experienced only 51% of the precipitation recorded in the 2010 growing season (Table 1). The distribution of precipitation over the course of the growing season in 2010 and 2011 is presented in Figure S3. A recent study has shown that drought conditions were associated with increased concentrations of aliphatic GS in *Brassica juncea* without a reduction in leaf biomass yield [38]. Thus, drought conditions in 2011 may have increased GS concentrations in both kale cultivars, although the GS increased was species specific.

### Effect of MeJA Treatment on Endogenous JA Concentrations

**Figure 2:**
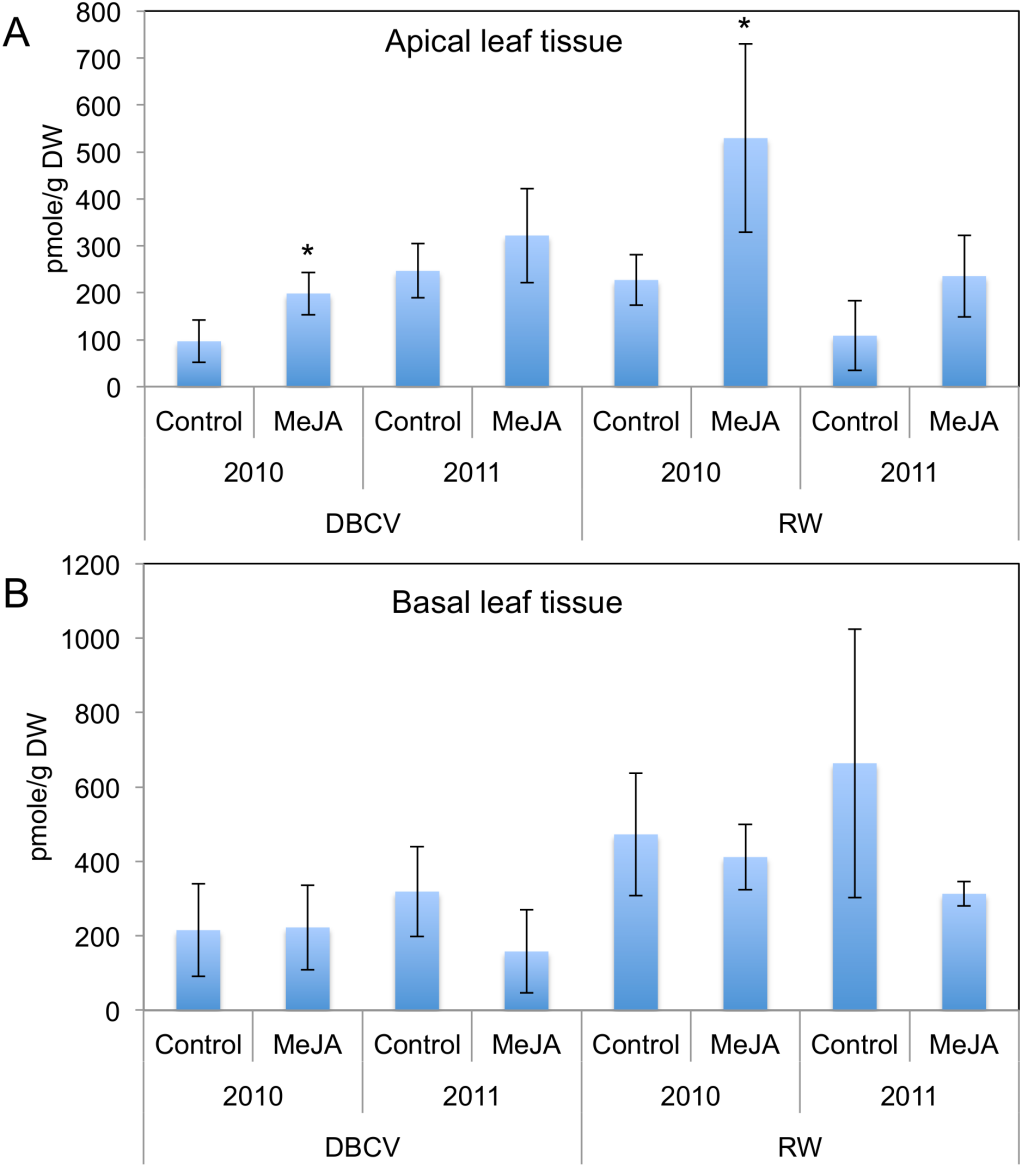
Effect of MeJA treatments on endogenous JA concentrations of apical and basal kale leaf tissue in two cultivars. Data are means ± SD (n = 3). *indicates significance at *P*≤0.05.

Endogenous JA concentrations in kale apical leaf tissue of two cultivars were significantly increased in 2010 by exogenous MeJA treatment but the treatment effect was not significant in 2011 (Figure 2A). Endogenous JA concentrations in apical leaves of control DBCV kale grown 2011 was significantly higher than control DBCV kale grown 2010. Endogenous JA has been observed to accumulate *in planta* under drought conditions [39]. The drought conditions in 2011 may have lead to the accumulation of endogenous JA which have could attenuate the effect of the exogenous MeJA treatment on DBCV. In contrast, endogenous JA concentrations in apical leaves of control RW kale harvested in 2011 was lower than control RW kale grown in 2010, implying endogenous JA concentration may be affected by other factors including insect activity and microenvironmental factors. Compared to DBCV, RW has relatively tender leaves and is more vulnerable to chewing insects. The RW cultivar displayed much more insect feeding activity by cabbage loopers [*Trichoplusia ni* (Hübner)] and flea beetles (*Phyllotreta cruciferae*) during the experiment (Figure S4). This insect activity and other environmental factors may compound drought effects on endogenous JA concentrations in RW and ultimately influence QR induction activity.

Unlike kale apical leaf tissue, MeJA treatment effects on basal leaf tissue did not have a significant influence on endogenous JA concentrations (Figure 2B). The concentration of endogenous JA in basal leaf tissues was higher than in apical leaf tissue, which may be related with insect feeding activity since basal leaves displayed greater insect damage. Reduction of endogenous JA in MeJA treatment groups in basal leaf tissue compared to control groups may be related with JA transport to apical leaf tissue in response to exogenous MeJA treatment. A recent study revealed that JA translocates from local damaged leaves systemically to other leaves in *Nicotiana tabacum* [40]. In another study, after radioactive JA application to one basal leaf, younger, apical leaves contained the most of total radioactivity in potato plants [41]. Higher JA accumulation in apical leaf tissue responding to exogenous MeJA may be related protection of younger tissues more important for plant reproduction and survival.

### Effect of MeJA Treatment on Myrosinase Activity

Previously we reported that MeJA treatment enhanced myrosinase gene expression and enzyme activity using greenhouse grown broccoli [26]. Unlike this earlier study with broccoli, there was no consistent response in myrosinase activity induced by MeJA treatments (Figure S5). This difference may be due to a tissue- (vegetative versus reproductive) or species-specific pattern of response. Myrosinase activity of field grown kale might also have been influenced by field biotic and abiotic factors, which also can change enzyme activity. There was significantly greater myrosinase activity in apical versus basal leaf tissue within each cultivar. In our previous research, we demonstrated a MeJA induced increase in myrosinase activity of greenhouse grown broccoli florets also contributed to QR inducing activity. However, in our two kale cultivars total myrosinase activity was observed to increase with MeJA treatment about 30% only in the apical leaf tissue of DBCV (2011 year). Increases were not seen in basal leaf tissue of either cultivar or in the apical leaf tissue of RW.

### Effect of MeJA Treatment on GS Hydrolysis Products Concentrations

Only sulforaphane was significantly increased in both apical and basal leaf tissue of the RW cultivar by MeJA treatment over two years. Increased concentrations of other hydrolysis products were not consistently observed in all samples over two years (Table 3). Despite significant increases in glucobrassicin, I3C and DIM hydrolysis product concentrations in kale extracts were relatively low. I3C has been reported to be highly instable [42] and will react with other substrates generating by-products by condensation with ascorbic acid or through oligomerization [12]. Following hydrolysis of the parent GS, relatively higher levels of NI3C were observed than I3C. According to previous research ascorbigen is more unstable than neoascorbigen [43]. I3C may be less stable than NI3C.

**Table 3:** Hydrolysis product composition of apical and basal leaf tissues with or without MeJA treatment from two kale cultivars over two years.

Apical tissue	––––––––DBCV––––––––				––––––––RW––––––––			
	2010		2011		2010		2011	
(µmol/g DW)	Control	MeJA	Control	MeJA	Control	MeJA	Control	MeJA
I3C	2.27	1.68^ns^	1.69	2.35*	0.92	1.05^ns^	1.35	2.74*
DIM	0.32	0.35^ns^	0.29	0.25^ns^	0.20	0.16^ns^	0.13	0.13^ns^
NI3C	1.11	1.30^ns^	0.73	1.37*	2.91	3.08^ns^	2.79	2.78^ns^
NeoASG	0.81	1.04^ns^	0.55	0.89^ns^	1.63	1.12^ns^	0.88	1.45*
Sulforaphane	0.05	0.09^ns^	0.07	0.06^ns^	0.28	0.51*	0.58	0.84*
**Basal tissue**	**––––––––DBCV––––––––**				**––––––––RW––––––––**			
	**2010**		**2011**		**2010**		**2011**	
**(µmol/g DW)**	**Control**	**MeJA**	**Control**	**MeJA**	**Control**	**MeJA**	**Control**	**MeJA**
I3C	0.70	0.93*	1.00	0.92^ns^	0.80	0.97^ns^	1.61*	1.11
DIM	0.14	0.26*	0.10	0.14^ns^	0.38*	0.10	0.25	0.12^ns^
NI3C	0.56	0.87*	0.30	0.38^ns^	1.07	1.61*	0.74	0.96^ns^
NeoASG	0.11	0.10^ns^	0.16	0.16^ns^	0.14	0.18^ns^	0.70*	0.31
Sulforaphane	0.01	0.05^ns^	0.01	0.03^ns^	0.00	0.08*	0.00	0.02*

Student T-tests were conducted to determine significance between control and MeJA treatment at *P*≤0.05. ^ns^ and * indicate non-significance and significance at *P*≤0.05, respectively.

Data are mean value from triplicates.

### Correlation Analysis between Intact GS or Hydrolysis Products, and Myrosinase activity

In order to elucidate the most active QR induction hydrolysis product in MeJA treated kale leaf tissue, correlation analysis was conducted between QR inducing activity and GS and GS hydrolysis product concentrations (Table 4). QR inducing activity significantly correlated with glucobrassicin (*r* = 0.747, *P* = 0.001) and I3C (*r* = 0.800, *P*<0.001). However, there was no significant correlation between myrosinase activity and QR inducing activity, which suggests QR was influenced by hydrolysis products of MeJA induced GS. This suggests that myrosinase was not a limiting factor in QR inducing activity in field grown kales. Since there is a significant difference in endogenous JA concentration between apical and basal leaf tissue, correlation analyses were conduced separately. There was significant positive correlation (*r* = 0.849, *P* = 0.008) between endogenous JA and neoglucobrassicin within apical leaf tissue. This suggests that increased endogenous JA levels induced by exogenous MeJA treatment not only stimulates glucobrassicin biosynthesis but also promotes GS side chain modification from glucobrassicin to neoglucobrassicin. A previous study revealed a positive correlation between pupal mass and development time of *Pieris brassicae* and foliar GS composition, of which levels of neoglucobrassicin appeared to be the most important [44]. This suggests that the side chain modification from glucobrassicin to neoglucobrassicin with increased endogenous JA may be related with insect herbivore defense. However, intact GS only have bioactivity after hydrolysis by myrosinase. There were significant correlations between total myrosinase activity and GS hydrolysis products including I3C (*r* = 0.724, P = 0.002), NeoASG (*r* = 0.546, P = 0.029), and sulforaphane (*r* = 0.541, P = 0.031). Although MeJA treatment did not significantly increase total myrosinase activity in kale leaves, both GS concentration and total myrosinase activity in apical leaf tissue were observed to be higher than in basal leaf tissue. These correlations imply that these GS hydrolysis products may be closely related with insect defense in apical leaf tissue.

**Table 4:** Correlation analysis between intact GS, GS hydrolysis products and QR inducing activity from apical and basal leaf tissue extracts from 250 µM MeJA treated two different kale cultivars over two years.

Variable	1	2	3	4	5	6	7	8	9	10	11
1. Glucoraphanin											
2. Glucobrassicin	0.120										
3. Gluconasturtiin	**0.726**	**0.561**									
4. Neoglucobrassicin	**0.500**	**0.731**	**0.914**								
5. QR	0.305	**0.747**	0.415	0.431							
6. I3C	0.203	**0.627**	0.358	0.422	**0.800**						
7. DIM	−0.374	0.308	−0.209	−0.067	0.042	0.227					
8. NI3C	**0.810**	0.330	**0.880**	**0.767**	0.176	0.209	−0.199				
9. NeoASG	**0.584**	**0.590**	**0.802**	**0.754**	0.381	**0.548**	0.075	**0.788**			
10. Sulforaphane	**0.879**	0.325	**0.907**	**0.742**	0.361	0.382	−0.342	**0.853**	**0.682**		
11. Endogenous JA	−0.138	0.496	0.575	**0.849**	0.005	−0.502	−0.262	0.294	0.153	0.157	
	(−0.188)	(−0.672)	**(**−0.489**)**	(0.247)	(−0.371)	(−0.022)	(0.442)	(0.362)	(**0.775**)	(−0.320)	
12. Myrosinase	**0.606**	0.391	0.402	0.333	0.433	**0.724**	0.110	0.439	**0.546**	**0.541**	**−0.123**

Bold values indicate significant correlations among variables from apical and basal leaf tissue extracts based on the Pearson’s correlation at *P*≤0.05 (n = 16). Upper and bottom values in endogenous JA row indicate correlation coefficients from apical and basal leaf tissue extracts, respectively (n = 8).

### I3C as QR inducer in kale leaf tissue

Using different concentrations of commercial I3C, the CD value for I3C was observed to be 230 µM (Figure S6), which is a relatively weak QR induction agent compared to sulforaphane (0.2 µM), 7–Methylsulfinylheptyl isothiocyanate (0.2 µM), PEITC (5 µM), and brassinin (4 µM) [17], . Previously we reported that the QR CD value of NI3C was 35 µM and neoascorbigen was 38 µM from broccoli extracts [21]. Despite the significantly increased amount of NI3C and neoascorbigen, their contribution to enhanced QR inducing activity was relatively small. The CD value of I3C does not fully explain the increased QR activity from kale leaf tissue extracts (Figure 1).

### MeJA Dose Dependent Induced GS and QR Activity in Kale Leaf Tissue

**Figure 3:**
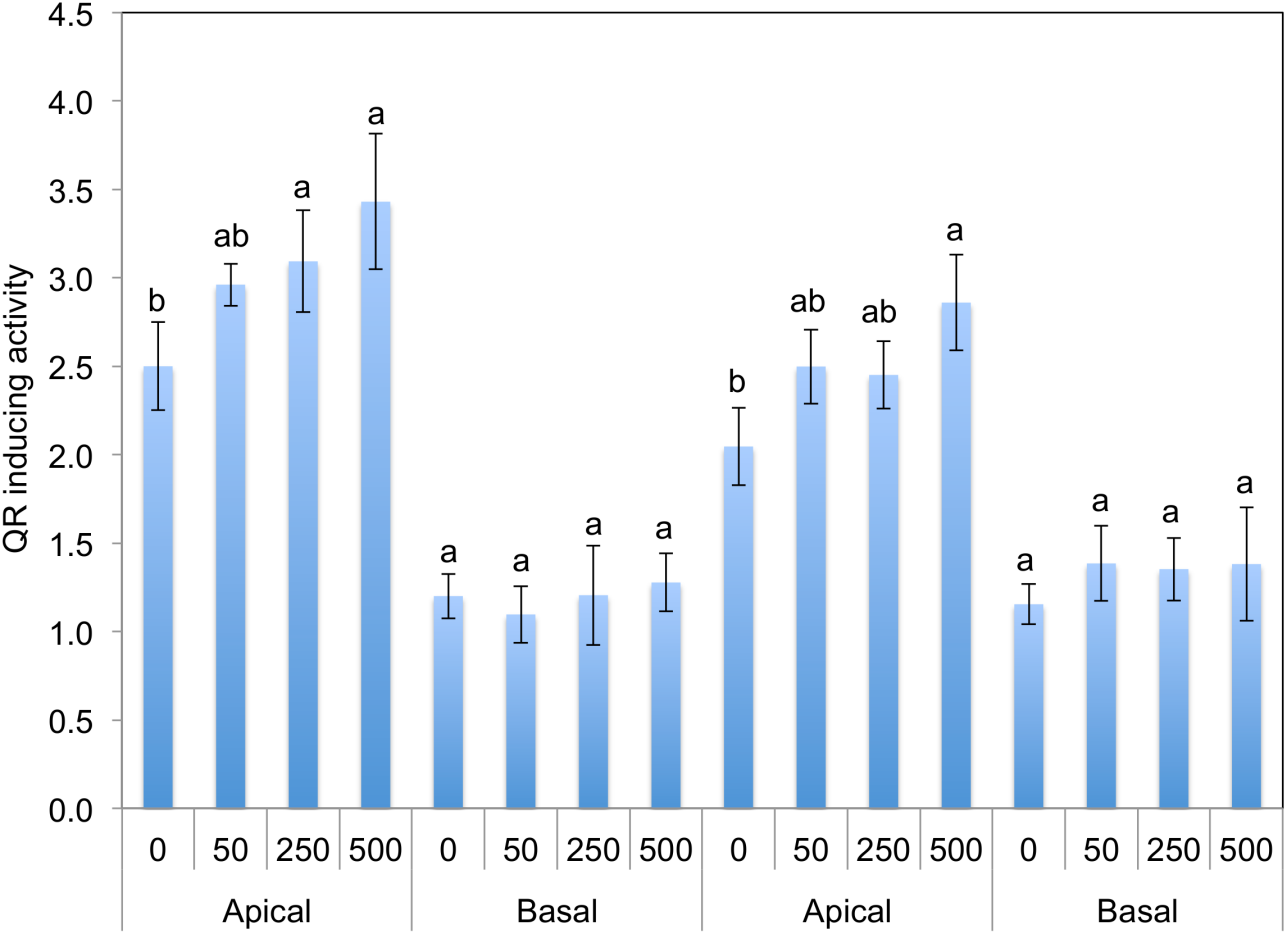
QR inducing activity from kale leaf tissue samples sprayed with varying concentrations of MeJA (0, 50, 250, and 500 µM). Different letters indicate significant differences among treatments based on Fisher’s LSD test at *P*≤0.05. A: QR activity, B: GS profiles, and C: hydrolysis product profiles.

To further evaluate the association between induction of QR activity and GS concentrations in kale leaves tissues, a second experiment was conducted where different MeJA concentrations (0, 50, 250, and 500 µM) were applied to two kale cultivars as described above. As concentrations of MeJA treatment increased GS tissue concentrations (glucobrassicin and neoglucobrassicin), QR activity was increased in apical leaf extracts of both kale cultivars (Figure 3, Table 5). In addition, MeJA treatment significantly increased NI3C and NeoASG in apical leaf tissue of both kale cultivars (Table 6). Although there was dose dependent increase in I3C by MeJA treatment from apical leaf tissue of the RW cultivar, DBCV kale showed a reduction in I3C concentrations in response to MeJA treatment (Table 6). MeJA treatment not only changes GS biosynthesis but also hydrolysis related gene expression [26]. Although higher apical leaf tissue indolyl GS hydrolysis product concentrations were found in RW compared to DBCV (Table 6), QR induction activity by RW apical leaf tissue was relatively low (Figure 3). The low concentration of I3C in kale leaf tissue may be related with very low stability or its condensation/oligomerization [12], [42]. Other hydrolysis products of glucobrassicin such as di(indol-3-yl)methane (DIM), brassinin, or 2,3-bis(indol-3-ylmethyl)-indole (TIR) which can induce QR activity at lower CD values [17], [19] may also play an important role in QR induction in kale than I3C.

**Table 5:** GS concentrations from kale leaf tissue samples sprayed with varying concentrations of MeJA (0, 50, 250, and 500 µM).

DBCV	––––––-Apical––––––-				––––––-Basal––––––-			
(µmol/g DW)	0	50	250	500	0	50	250	500
Glucoiberin	9.62 a	11.39 a	8.46 a	9.25 a	0.71 a	0.73 a	0.77 a	0.71 a
Progoitrin	1.03 a	0.39 a	0.29 a	0.22 a	0.05 a	0.03 a	0.06 a	0.02 a
Glucoraphanin	0.07 a	0.07 a	0.08 a	0.08 a	0.00 a	0.00 a	0.00 a	0.01 a
Gluconapin	1.28 a	1.35 a	1.20 a	1.19 a	1.22 a	1.10 a	0.99 a	0.82 a
Glucobrassicin	21.31 b	21.42 b	29.73 a	31.92 aa	2.53 a	2.96 a	3.24 a	3.47 a
Gluconasturtiin	0.97 a	1.50 a	1.29 a	0.86 a	0.47 a	0.68 a	0.54 a	0.47 a
Neoglucobrassicin	4.51 b	8.33 a	9.21 a	10.49 a	0.98 a	1.08 a	0.88 a	1.02 a
**RW**	**––––––-Apical––––––-**				**––––––-Basal––––––-**			
**(µmol/g DW)**	**0**	**50**	**250**	**500**	**0**	**50**	**250**	**500**
Glucoiberin	0.00 a	0.03 a	0.03 a	0.03 a	0.00 a	0.00 a	0.00 a	0.00 a
Progoitrin	31.60 a	31.48 a	26.67 a	29.17 a	2.64 a	2.43 a	2.23 a	2.16 a
Glucoraphanin	5.48 a	4.24 a	3.39 a	3.76 a	0.08 a	0.04 a	0.37 a	0.07 a
Gluconapin	1.22 a	1.43 a	1.20 a	0.92 a	1.32 a	1.02 a	0.67 a	1.27 a
Glucobrassicin	5.93 b	14.83 a	15.86 a	15.81 a	0.85 b	0.93 b	1.24 ab	1.65 a
Gluconasturtiin	2.05 a	2.80 a	2.72 a	2.68 a	0.48 a	0.33 a	0.32 a	0.32 a
Neoglucobrassicin	6.17 b	12.30 a	12.61 a	13.58 a	2.03 b	1.83 b	2.57 ab	3.21 a

Different letters indicate significant differences among treatments based on Fisher’s LSD test at *P*≤0.05. Data are mean value from triplicates.

**Table 6:** GS hydrolysis product concentrations from kale leaf tissue samples sprayed with varying concentrations of MeJA (0, 50, 250, and 500 µM).

DBCV	––––––-Apical––––––-				––––––-Basal––––––-			
(µmol/g DW)	0	50	250	500	0	50	250	500
I3C	1.32 a	1.20 a	0.65 b	0.60 b	0.17 a	0.21 a	0.13 a	0.12 a
DIM	0.18 a	0.13 a	0.14 a	0.14 a	0.09 a	0.10 a	0.11 a	0.10 a
NI3C	1.71 b	2.54 a	2.83 a	2.88 a	0.41 b	0.69 a	0.55 b	0.50 b
NeoASG	0.42 b	0.63 a	0.53 a	0.44 a	-	-	-	-
Sulforaphane	0.01 a	0.01 a	0.01 a	0.01 a	-	-	-	-
**RW**	**––––––-Apical––––––-**				**––––––-Basal––––––-**			
**(µmol/g DW)**	**0**	**50**	**250**	**500**	**0**	**50**	**250**	**500**
I3C	1.35 b	1.55 b	1.86 a	1.86 a	0.09 b	0.45 a	0.44 a	0.23 b
DIM	0.15 a	0.15 a	0.19 a	0.17 a	0.09 a	0.09 a	0.10 a	0.09 a
NI3C	2.83 b	4.00 a	4.15 a	4.50 a	0.88 b	0.98 b	1.49 a	1.78 a
NeoASG	0.44 b	0.44 b	0.65 a	0.96 a	-	-	-	-
Sulforaphane	0.54 b	0.60 a	0.71 a	0.62 a	0.02 a	0.02 a	0.03 a	0.03 a

Different letters indicate significant differences among treatments based on Fisher’s.

LSD test at *P*≤0.05. Data are mean value from triplicates.

### Correlation Analysis of GS, GS Hydrolysis Products and QR with Varying Treatment Concentrations of MeJA

Correlation of QR activity of the two kale cultivars over the two seasons were significant for gluconasturtiin (*r* = 0.888, *P*<0.001), glucobrassicin (*r* = 0.671, *P* = 0.001), and neoglucobrassicin (*r* = 0.980, *P*<0.001). The GS hydrolysis products I3C (*r* = 0.856, <0.001), DIM (*r* = 0.788, *P*<0.001), NI3C (*r* = 0.974, *P*<0.001), NeoASG (*r* = 0.918, *P*<0.001) and sulforaphane (*r* = 0.770, *P*<0.001) also correlated with QR activity (Table 7). Sulforaphane is the predominant QR induction agent in MeJA treated broccoli extracts [21], [26]. Similarly, sulforaphane may play an important role to induce QR activity in RW leaf extracts as in broccoli extracts. In case of DBCV, this data suggests that the combination of I3C and its derivatives, NI3C and NeoASG induction contributed to enhanced QR activity of kale leaf tissue extracts. Since correlation analysis does not necessarily imply causation, further research is needed to address which compound or compounds are dominating QR induction in kale leaf tissue.

**Table 7:** Correlation analysis between intact GS, GS hydrolysis product and QR activity from kale leaf tissue across two kale cultivars sprayed with varying concentrations of MeJA (0, 50, 250, and 500 µM).

Variable	1	2	3	4	5	6	7	8	9
1. Glucoraphanin									
2. Glucobrassicin	0.081								
3. Gluconasturtiin	**0.858**	0.444							
4. Neoglucobrassicin	**0.621**	**0.734**	**0.872**						
5. QR	**0.704**	**0.671**	**0.888**	**0.980**					
6. I3C	**0.768**	**0.504**	**0.920**	**0.841**	**0.856**				
7. DIM	**0.603**	**0.682**	**0.808**	**0.795**	**0.788**	**0.904**			
8. NI3C	**0.731**	**0.620**	**0.903**	**0.976**	**0.974**	**0.886**	**0.797**		
9. NeoASG	**0.581**	**0.742**	**0.845**	**0.914**	**0.918**	**0.890**	**0.867**	**0.897**	
10. Sulforaphane	**0.947**	0.133	**0.917**	**0.719**	**0.770**	**0.830**	**0.675**	**0.806**	**0.649**

Bold values indicate significant correlations based on the Pearson’s correlation at *P*≤0.05.

In a previous study we reported that MeJA treatment significantly increased QR inducing activity in cauliflower and broccoli, which was primarily associated with glucoraphanin and its hydrolysis product sulforaphane [21], [23]. It is interesting that MeJA treatment can significantly increase QR inducing activity in the DBCV cultivar, which does not have high glucoraphanin concentrations in comparison to RW. This is at least partially due to increases in the flavonoid phenolics. In addition, apical leaf tissue of both RW and DBCV kale have significantly higher QR inducing activity and GS levels than basal leaves. These results will provide helpful information for kale production with enhanced consumer health promoting properties.

## Supporting Information

Figure S1 **QR inducing activity of 70% myrosinase-inactivated methanol extracts from different kale leaf tissues with or without MeJA treatment from two kale cultivars over two years.**
(TIF)

Figure S2 **Optimum harvest date for MeJA treated kale leaf tissue based on the GS concentrations. Data are means (n = 3).**
(TIF)

Figure S3 **Precipitation information in 2010 and 2011.**
(TIF)

Figure S4 **Visual insect damage differences of two kale cultivars in 2010.**
(TIF)

Figure S5 **Myrosinase activity of different kale leaf tissue samples with or without MeJA treatment from two kale cultivars over two years.**
(TIF)

Figure S6 **QR inducing activity of indole-3-carbinol (I3C).** Seven different concentrations from 3.9 to 250 µM were tested using QR assay to determine CD value of I3C. Data are means ± SD (n = 3).
(TIF)
