# Supplementary figures and images for: Exogenous Methyl Jasmonate Treatment Increases Glucosinolate Biosynthesis and Quinone Reductase Activity in Kale Leaf Tissue
Source: PLoS One. 2014 Aug 1;9(8):e103407. doi: 10.1371/journal.pone.0103407 (PMC4118879; doi:10.1371/journal.pone.0103407)

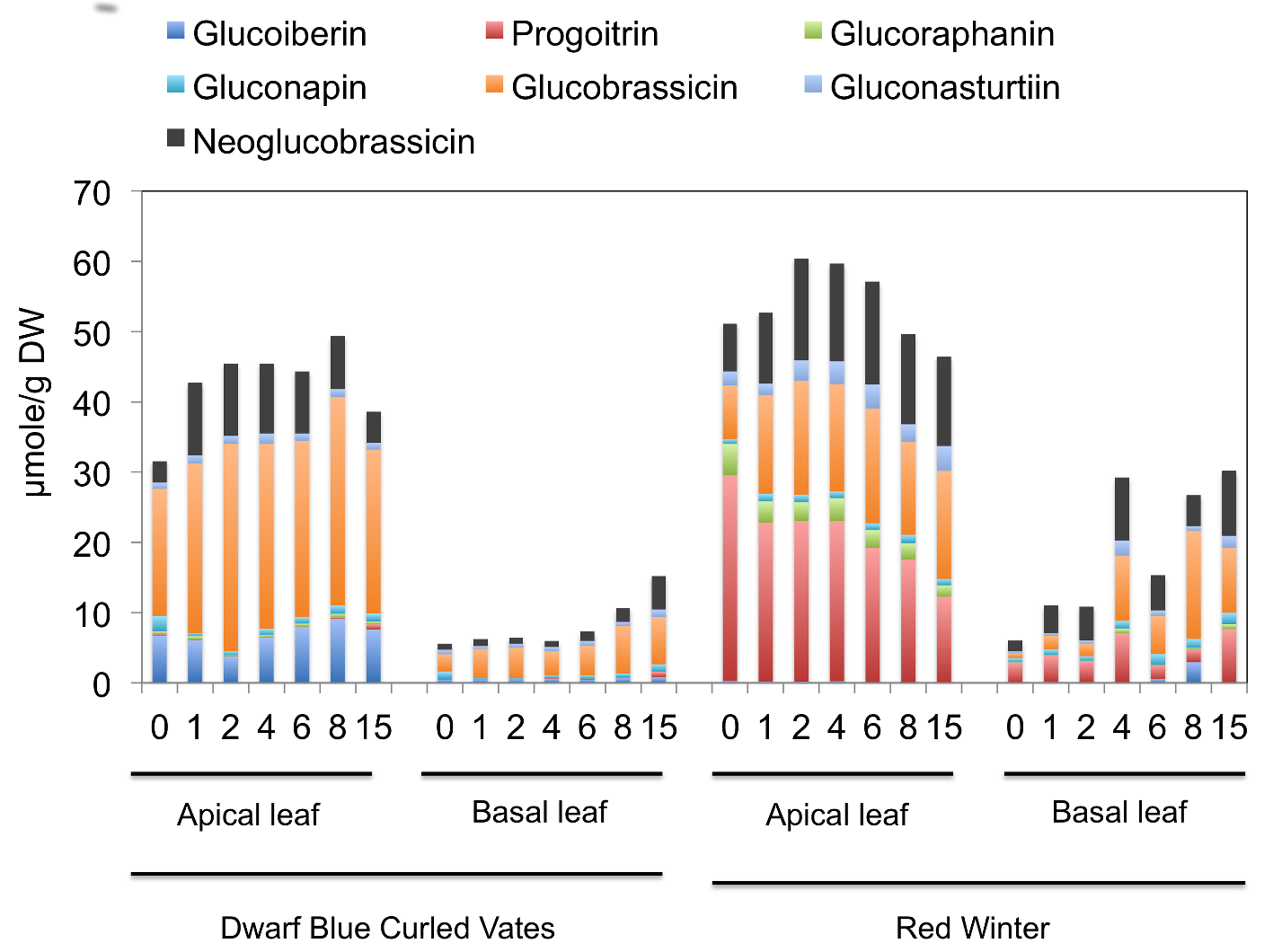

Supplement: Figure S1 — QR inducing activity of 70% myrosinase-inactivated methanol extracts from different kale leaf tissues with or without MeJA treatment from two kale cultivars over two years. (TIF) [file pone.0103407.s001.tif]

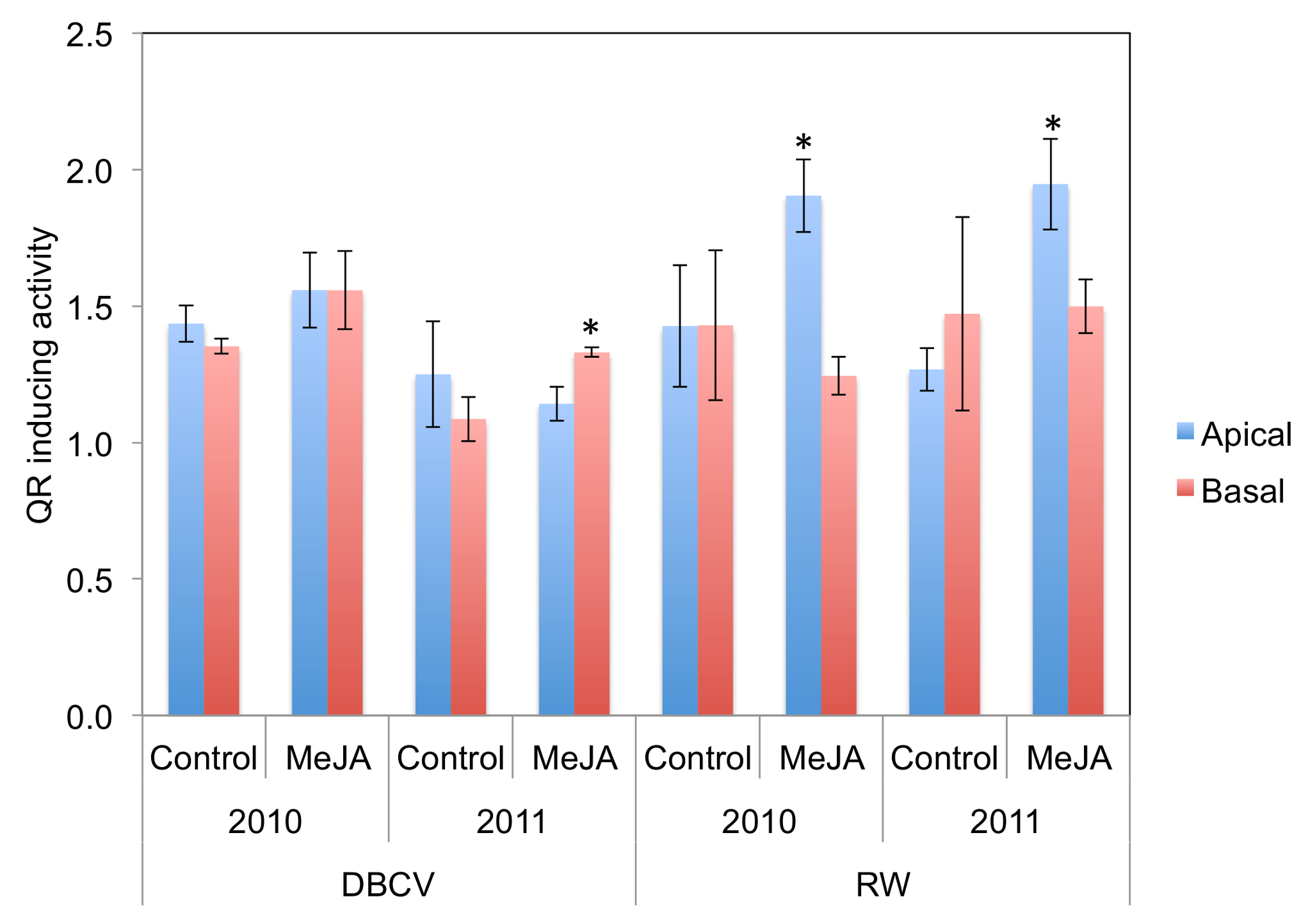

Supplement: Figure S2 — Optimum harvest date for MeJA treated kale leaf tissue based on the GS concentrations. Data are means (n = 3). (TIF) [file pone.0103407.s002.tif]

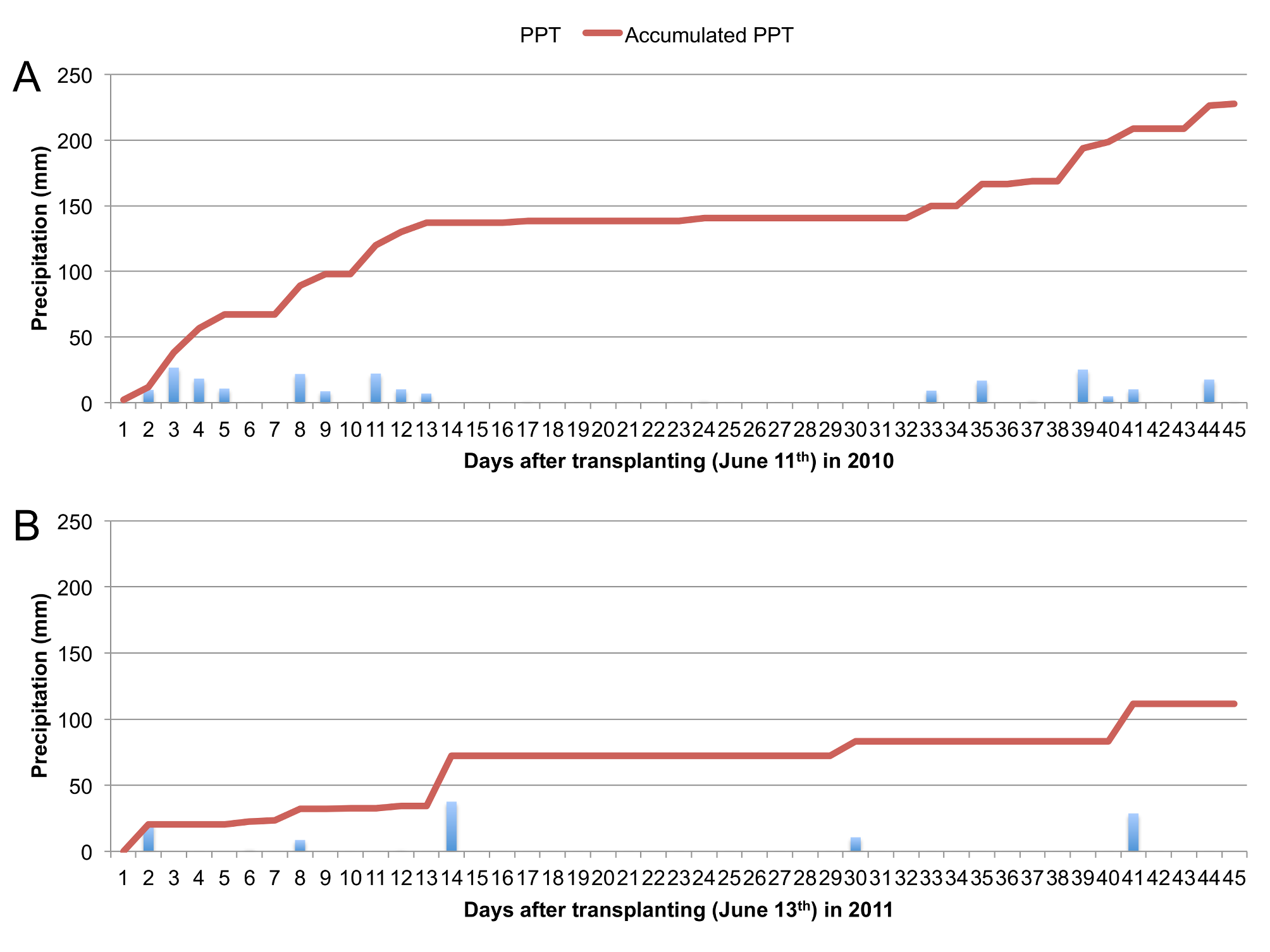

Supplement: Figure S3 — Precipitation information in 2010 and 2011. (TIF) [file pone.0103407.s003.tif]

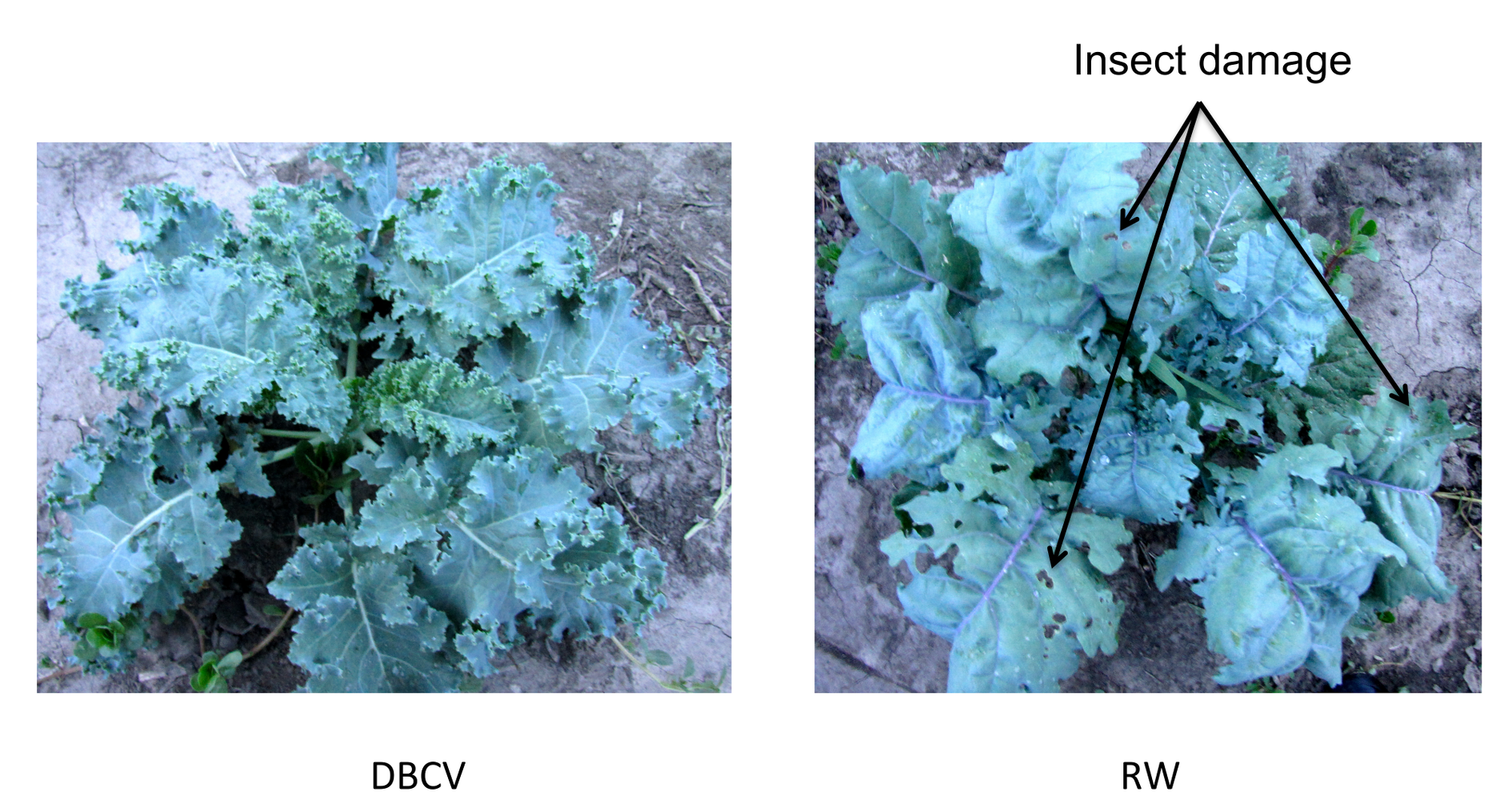

Supplement: Figure S4 — Visual insect damage differences of two kale cultivars in 2010. (TIF) [file pone.0103407.s004.tif]

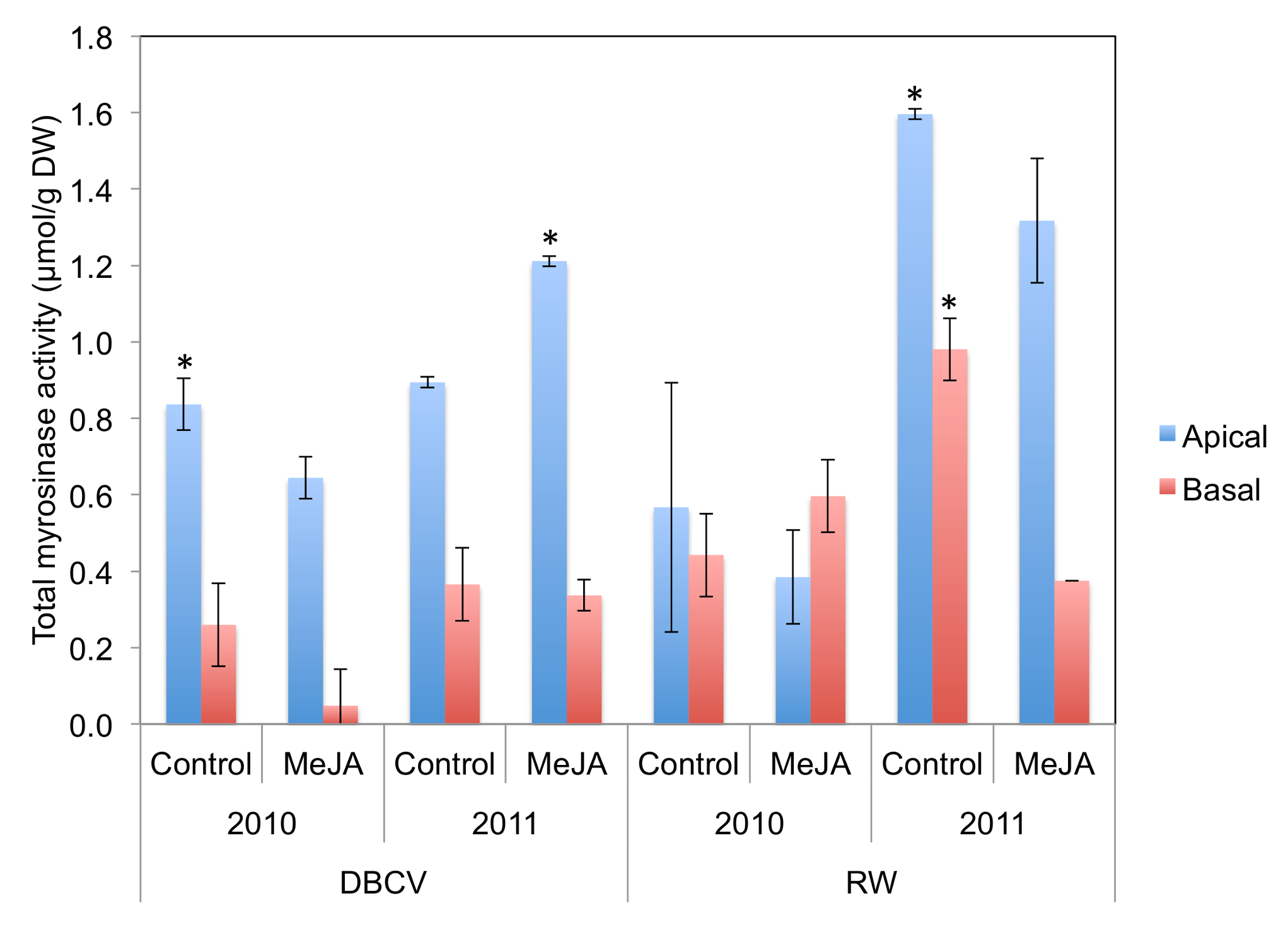

Supplement: Figure S5 — Myrosinase activity of different kale leaf tissue samples with or without MeJA treatment from two kale cultivars over two years. (TIF) [file pone.0103407.s005.tif]

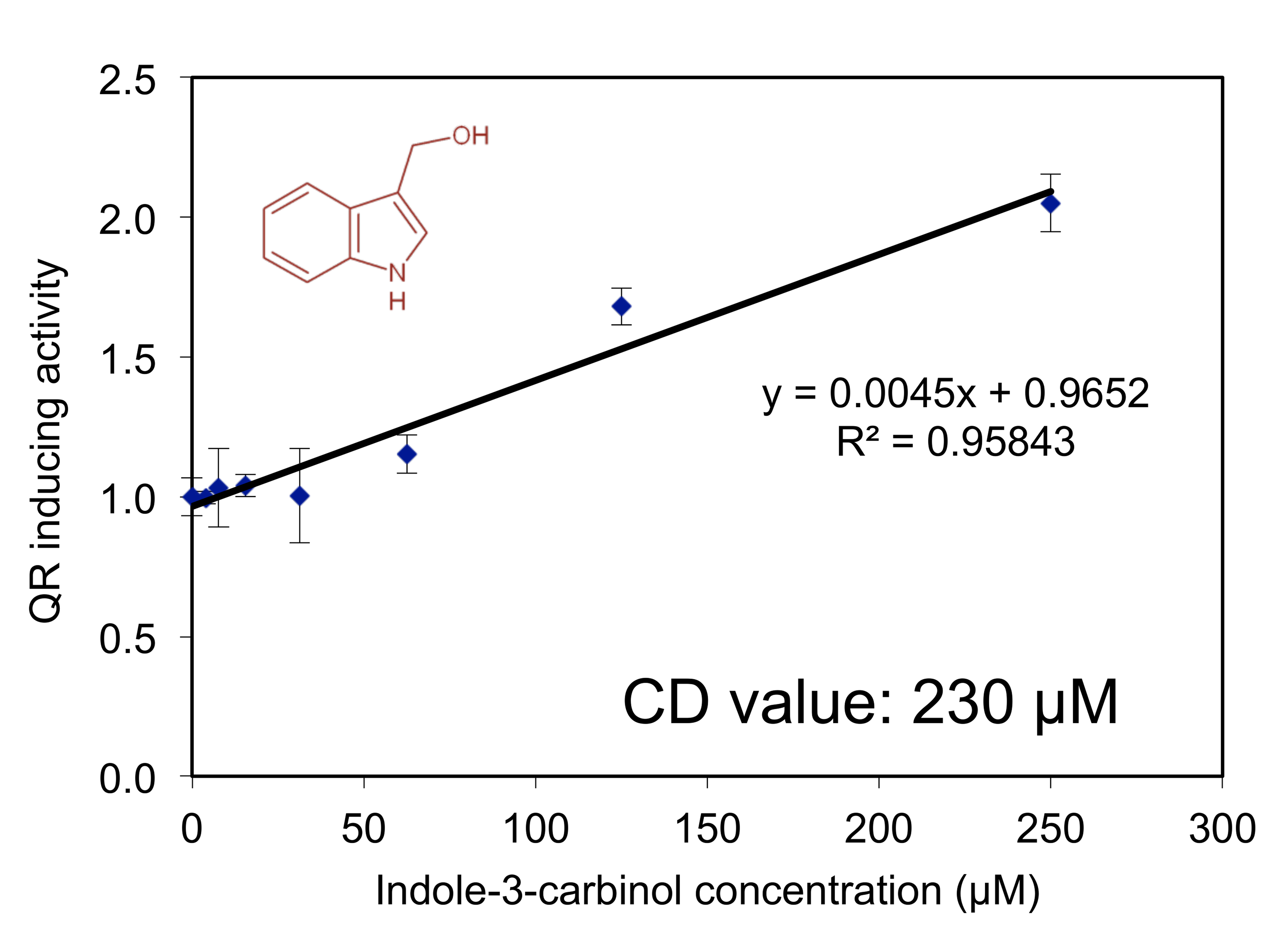

Supplement: Figure S6 — QR inducing activity of indole-3-carbinol (I3C). Seven different concentrations from 3.9 to 250 µM were tested using QR assay to determine CD value of I3C. Data are means ± SD (n = 3). (TIF) [file pone.0103407.s006.tif]
